# Complete Genome Sequence of *bla*_CTX-M-27_-Encoding *Escherichia coli* Strain H105 of Sequence Type 131 Lineage C1/H30R

**DOI:** 10.1128/genomeA.00736-17

**Published:** 2017-08-03

**Authors:** Hiren Ghosh, Boyke Bunk, Swapnil Doijad, Judith Schmiedel, Linda Falgenhauer, Cathrin Spröer, Can Imirzalioglu, Jörg Overmann, Trinad Chakraborty

**Affiliations:** a Institute of Medical Microbiology, Justus Liebig University, Giessen and German Center for Infection Research (DZIF), Partner site Giessen-Marburg-Langen, Giessen, Germany; b Leibniz Institute DSMZ-German Collection of Microorganisms and Cell Cultures, German Center for Infection Research (DZIF), Partner site Hannover-Braunschweig, Braunschweig, Germany

## Abstract

*Escherichia coli* sequence type 131 (ST131) is the most frequent antimicrobial-resistant lineage of *E. coli*, propagating extended-spectrum β-lactamases (ESBL) worldwide. Recently, an alarming rate of increase in isolates of the sublineage C1/H30R-*bla*_CTX-M-27_ of ST131 in geographically distant countries was reported. Here, we present the complete genome sequence of the ST131 sublineage C1/H30R *E. coli* isolate harboring *bla*_CTX-M-27_ from Germany.

## GENOME ANNOUNCEMENT

We sequenced the *Escherichia coli* isolate H105, a sequence type 131 (ST131) C1/H30R extended-spectrum β-lactamase (ESBL)-producing isolate obtained in 2010 from a vaginal swab sample of an individual from Germany. The isolate harbors a *bla*_CTX-M-27_ allele and represents an early isolate with this genotype combination in our collection. As reported in Japan (1) and in France (2), there is currently a strong surge in the number of ST131-C1/H30R-*bla*_CTX-M-27_ isolates detected in Germany, particularly in the past 3 years, suggesting an ongoing shift in CTX-M alleles associated with ST131 infections.

For the whole-genome sequencing, genomic DNA was isolated using a PureLink genomic DNA kit (Invitrogen, Darmstadt, Germany) from an overnight culture grown at 37°C in LB medium. Short-read sequencing was performed on an Illumina MiSeq machine (Illumina, the Netherlands) using a Nextera XT library with MiSeq v3 (2 × 300 bp) reagent kit. Single-molecule real-time sequencing (SMRT) was conducted using the PacBio RSII system (Pacific Biosciences, USA).

*De novo* genome assembly of 59,447 PacBio reads with an average read length of 10,355 bp was performed using the “RS_HGAP_Assembly.3” included in the SMRT Portal version 2.3.0. Subsequently, Illumina short reads were mapped onto the assembled sequences in order to obtain a highly accurate genome with QV60 final quality. The chromosome was adjusted to *dnaA* as the first gene. Annotation was performed both using Prokka 1.10 (3) and the NCBI Prokaryotic Genome Annotation Pipeline (PGAP) (http://www.ncbi.nlm.nih.gov/genomes/static/Pipeline.html). Antimicrobial resistance genes and prophages were identified using Resfinder and PHAST, respectively (4, 5).

The assembly resulted in a closed, circular chromosomal contig of 4,978,342 bp and a plasmid of 134,499 bp, designated the plasmid pH 105. The chromosome of *E. coli* H105 exhibited a 50.7% G+C content, and harbored 4,635 open reading frames, 87 tRNAs, 15 rRNAs, and 6 intact prophage regions. *In silico* analysis revealed that *E. coli* H105 belongs to the sequence type ST131, serotype O25:H4, and phylogroup B2. The genome of H105 includes multiple chromosomally encoded virulence genes such as for iron uptake (yersiniabactin, aerobactin, *sitABC*), serum resistance (*iss, traT*), autotransporter proteases (*sat, pic*), and the postsegregational killing system *ccdA/ccdB*.

The plasmid pH105 is a multireplicon plasmid, depicting IncFIA, FIB, and FII replicons with the pMLST type F1:A2:B20 (6). The ESBL gene *bla*_CTX-M-27_ encoded on pH105 confers resistance to extended-spectrum β-lactams. In addition, pH105 also carries genes conferring resistance to aminoglycosides (*aadA5, strA, strB*), macrolides [*mph*(A)], tetracyclines [*tet*(B)], sulfonamide (*sul1, sul2*), and trimethoprim (*dfrA17*).

The sequence of H105 represents the first complete genome of the *bla*_CTX-M-27_-encoding *E. coli* ST131 of the lineage C1/H30R. The high-quality genome of H105 will serve as a valuable resource for comparative studies on epidemiology of globally emerging ST131-C1/H30R-*bla*_CTX-M-27_.

### Accession number(s).

The complete genome sequence of *E. coli* H105 has been deposited in GenBank under GenBank accession no. CP021454 (chromosome) and CP021871 (plasmid).

## References

[1] Matsumura Y, Pitout JDD, Gomi R, Matsuda T, Noguchi T, Yamamoto M, Peirano G, DeVinney R, Bradford PA, Motyl MR, Tanaka M, Nagao M, Takakura S, Ichiyama S 2016 Global *Escherichia coli* sequence type 131 clade with blaCTX-M-27 gene. Emerg Infect Dis 22:1900–1907. doi:10.3201/eid2211.160519. 27767006 PMC5088012

[2] Birgy A, Bidet P, Levy C, Sobral E, Cohen R, Bonacorsi S 2017 CTX-M-27-producing *Escherichia coli* of sequence type 131 and clade C1-M27, France. Emerg Infect Dis 23:885. doi:10.3201/eid2305.161865. PMC5403054 28418829

[3] Seemann T 2014 Prokka: rapid prokaryotic genome annotation. Bioinformatics 30:2068–2069. doi:10.1093/bioinformatics/btu153. 24642063

[4] Zankari E, Hasman H, Cosentino S, Vestergaard M, Rasmussen S, Lund O, Aarestrup FM, Larsen MV 2012 Identification of acquired antimicrobial resistance genes. J Antimicrob Chemother 67:2640–2644. doi:10.1093/jac/dks261. 22782487 PMC3468078

[5] Arndt D, Grant JR, Marcu A, Sajed T, Pon A, Liang Y, Wishart DS 2016 PHASTER: a better, faster version of the PHAST phage search tool. Nucleic Acids Res 44:W16–W21. doi:10.1093/nar/gkw387. 27141966 PMC4987931

[6] Carattoli A, Zankari E, García-Fernández A, Voldby Larsen M, Lund O, Villa L, Møller Aarestrup F, Hasman H 2014 *In silico* detection and typing of plasmids using PlasmidFinder and plasmid multilocus sequence typing. Antimicrob Agents Chemother 58:3895–3903. doi:10.1128/AAC.02412-14. 24777092 PMC4068535

